# Gestational Intermittent Hypoxia Induces Mitochondrial Impairment in the Geniohyoid Muscle of Offspring Rats

**DOI:** 10.7759/cureus.25088

**Published:** 2022-05-17

**Authors:** Wirongrong Wongkitikamjorn, Jun Hosomichi, Eiji Wada, Hideyuki Maeda, Sirichom Satrawaha, Haixin Hong, Yukiko K Hayashi, Ken-ichi Yoshida, Takashi Ono

**Affiliations:** 1 Department of Orthodontic Science, Graduate School of Medical and Dental Sciences, Tokyo Medical and Dental University (TMDU), Tokyo, JPN; 2 Department of Orthodontics, Faculty of Dentistry, Chulalongkorn University, Bangkok, THA; 3 Department of Pathophysiology, Tokyo Medical University, Tokyo, JPN; 4 Department of Forensic Medicine, Tokyo Medical University, Tokyo, JPN; 5 Department of Stomatology, Shenzhen University General Hospital, Shenzhen, CHN

**Keywords:** offspring rat, masseter muscles, geniohyoid muscle, intermittent hypoxia, pregnancy, obstructive sleep apnea

## Abstract

Introduction

Gestational intermittent hypoxia (IH), a hallmark of obstructive sleep apnea during gestation, alters respiratory neural control and diaphragm muscle contractile function in the offspring. The geniohyoid (GH) muscle is innervated by the respiratory-related hypoglossal nerve and plays a role in tongue traction and suckling, motor behaviors that then give way to chewing. Here, we aimed to investigate the effects of gestational exposure to IH on the muscle development and metabolism of GH and masseter muscles in male offspring rats.

Materials and methods

Pregnant Sprague-Dawley rats were exposed to IH (3-min periods of 4-21% O_2_) for eight hours/day during gestational days 7-20. The GH and masseter muscles from 35-day-old male offspring (n = 6 in each group) were analyzed.

Results

Gestational IH induction reduced type IIA fiber size in the GH muscle of the offspring but not in the masseter muscle. Western blot analysis showed that gestational IH-induced significant downregulation of peroxisome proliferator-activated receptor (PPAR)-gamma coactivator 1-alpha (PGC1α) protein in the GH muscle but not in the masseter muscle. Moreover, optic atrophy 1 and mitofusin-2 proteins were decreased and mitochondrial fission 1 protein levels were increased in the GH muscle of the offspring exposed to gestational IH. Mitochondrial adenosine triphosphate (ATP) synthase subunit alpha and transcriptional factor A (TFAM) were decreased in the GH muscle post-gestational IH.

Conclusion

These findings suggest that gestational IH-induced impaired mitochondrial metabolism and alteration of oxidative myofibers of the GH muscle in the pre-adolescent offspring, but not the masseter muscle, owing to the susceptibility of GH muscular mitochondria to gestational IH.

## Introduction

Fetal development is susceptible to acute changes in maternal oxygen levels in rodents and humans [1,2]. Maternal intermittent hypoxia (IH), a hallmark of gestational obstructive sleep apnea (OSA), affects long-term postnatal development and increases the risk of cardiovascular and respiratory dysfunctions in the offspring [3]. Johnson et al. have shown that gestational IH increases susceptibility to neuroinflammation and alters respiratory motor control in the offspring [4]. Gestational IH also decreases the contractile function of the diaphragm muscle in offspring rats, which suggests that IH reduces hypoxic tolerance of the diaphragm muscle during postnatal development [5].

Respiratory neurons in the reticular formation project to the hypoglossal motor nucleus in the brainstem, which in turn transmits the respiratory drive signal to the genioglossus and geniohyoid (GH) muscles of the tongue [6] via the medial branch of the hypoglossal nerve. The GH muscle is also innervated by fibers joined from the first cervical nerve. Electromyographic studies of rodent models and humans revealed that hypoxia or hypercapnia increases the respiratory drive to hypoglossal motoneurons and tongue muscles [7]. The GH muscle has a role in suckling, a motor behavior that then gives way to chewing. Gestational IH may increase the impaired respiratory drive to hypoglossal motoneurons, which affects the growth and development of GH muscles in young offspring. Thus, respiratory motor control of the GH muscle casts doubt on whether gestational IH affects postnatal development of the GH muscle, thus contributing to suckling and feeding in young offspring.

Postnatal IH induces mitochondrial dysfunction with a significant decrease in peroxisome proliferator-activated receptor (PPAR)-gamma coactivator 1-alpha (PGC1α) in rat genioglossal muscle [8]. Mitochondria are extremely sensitive to environmental stress, such as hypoxia and ischemia-reperfusion, and mitochondrial dysfunction causes a progressive loss of muscle strength and fatigue resistance. Moreover, environmental stress frequently causes mitochondrial fusion and fission to maintain their functionality. PGC1α increases the expression of pro-fusion proteins, optic atrophy 1 (OPA1), mitofusin (MFN1), and MFN2; an increase in the levels of MFN1 and MFN2 enhances mitochondrial fusion. PGC1α also decreases the levels of the pro-fission protein guanosine triphosphatase dynamin-related protein 1 and mitochondrial fission 1 (FIS1), thus inhibiting mitochondrial fission in vitro [9,10]. However, the effects of gestational IH on postnatal development and metabolism of the GH muscle are unclear.

Ventilatory stimuli activate the jaw-closing muscles, as well as the genioglossus and GH muscles, to stabilize the mandible and upper airway patency in humans [11]. The hypoglossal nucleus contains motoneurons that innervate tongue muscles, while the motor trigeminal nucleus contains motoneurons that control jaw-closing muscles, including the masseter (MAS) muscles. Interneurons of the motor trigeminal nucleus are part of the lateral tegmental field projections to the hypoglossal nucleus [12]. Thus, the MAS muscle not only functions as the jaw-closing muscle, but its activity is also modulated by the respiratory inputs. However, the GH muscle shows respiratory-related cyclic activity even at rest [13], in contrast to the MAS muscle [14], and there may be some differences in physiological responses between the GH and MAS muscles as accessory muscles of respiration. The aim of this study was to investigate the effects of gestational exposure to chronic IH on the muscle development and metabolism of GH and MAS muscles in male offspring rats. In this study, we characterized different responses of the GH and MAS muscles to gestational IH in male offspring rats.

## Materials and methods

Experimental model

Six pregnant Sprague-Dawley rats (225-250 g) were randomly exposed to normoxia (N, n = 3) and IH (n = 3), at a rate of 20 cycles/hour (nadir, 4% oxygen; peak, 20% oxygen; 0% carbon dioxide) for eight hours/day during the 12-h “lights on” period, from gestation days 7 to 20, as previously described [15]. Blood oxygen saturation (SpO_2_) levels were measured using a pulse oximeter (MouseOx; STARR Life Sciences Corp., Oakmont, PA, USA) placed on the neck of pregnant rats during IH cycles at gestation day 20. Mother rats were given ad libitum access to food and water throughout the experiment. All pups from both groups were born naturally under normoxia (gestational normoxia with postnatal normoxia; N/N, prenatal intermittent hypoxia with postnatal normoxia; IH/N) and kept with their mothers until weaning. At the age of five weeks, we randomly chose male pups from each mother rat, and six pups per each group were anesthetized with isoflurane and euthanized.

The experimental procedures used in this study were approved by the Institutional Animal Care and Use Committee of Tokyo Medical University (ethics approval number: H31-0011).

Sample preparation and histological analyses

GH and MAS muscles were collected immediately from the euthanized male pups, frozen with isopentane in liquid nitrogen, and stored at −80 °C for histological analyses. Transverse 10 µm thick cryosections were stained with hematoxylin and eosin, modified Gomori trichrome, and nicotinamide adenine dinucleotide reductase (NADH) stains.

Immunohistochemistry

Transverse 8 µm-thick serial cryosections of the muscles were collected and blocked with 2% bovine serum albumin in phosphate-buffered saline. Each section was stained with primary antibodies against myosin heavy chain (MHC) type I, BA-F8, IIA, SC-71, IIX/D, 6H1, or IIB; BF-F3 (DSHB, Iowa City, IA, USA) (Table 1), with a muscle cell membrane with laminin at 37 °C for 80 min. For fiber size analysis, Alexa Fluor 488 anti-mouse and 568 anti-rabbit secondary antibodies (1:1000 dilution; Thermo Fisher Scientific, Waltham, MA, USA) were used for detection. For fiber type distribution staining, anti-mouse Immunoglobulin G2b Alexa Fluor 350, anti-mouse Immunoglobulin G1 Alexa Fluor 488, and anti-mouse IgM Alexa Fluor 555 (1:1000 dilution; Thermo Fisher Scientific, Waltham, MA, USA) were used for detection. All staining images were acquired using a fluorescence microscope (Zeiss, Oberkochen, Germany). The sections for fiber size analysis were captured using the IN Cell Analyzer 2200 imaging system for calculating the muscle fiber size (diameters in minor axis) with IN Cell Developer Toolbox software (GE Healthcare, Chicago, IL, USA). The basal membrane was detected by laminin staining to calculate fiber size, and each myosin heavy chain-positive fiber was automatically selected by intensity. Muscle fiber size was assessed by quantifying the short diameters on the cross-sectional images. Data were analyzed as frequency distributions by comparing the N/N and IH/N groups. The sections for fiber type distribution were analyzed using Image J software and evaluated as the fiber distribution ratio.

**Table 1:** Primary antibodies list. IHC: immunohistochemistry; WB: western blot; MHC: myosin heavy chain.

Antibody name	Target	Catalog number	Manufacturer	Application	Concentration	Host species
BA-F8	MHC type I	BA-F8	DSHB	IHC	1:50	Mouse
SC-71	MHC type IIA	SC-71	DSHB	IHC	1:600	Mouse
6H1	MHC type IIX/D	6H1	DSHB	IHC	1:50	Mouse
BF-F3	MHC type IIB	BF-F3	DSHB	IHC	1:100	Mouse
L9393	Laminin	L9393	Sigma-Aldrich	IHC	1:200	Rabbit
15H4C4	ATP5A1	ab14748	Abcam	WB	1:500	Mouse
6C5	GAPDH	ab8245	Abcam	WB	1:10000	Mouse
13798-1-AP	MFN1	13798-1-AP	Proteintech	WB	1:500	Rabbit
12186-1-AP	MFN2	12186-1-AP	Proteintech	WB	1:500	Rabbit
EPR2796	NDUFAF1	ab79826	Abcam	WB	1:500	Rabbit
NB110-55290	OPA1	NB110-55290	Novus Biologicals	WB	1:500	Rabbit
NBP1-04676	PGC1α	NBP1-04676	Novus Biologicals	WB	1:500	Rabbit
ab131607	TFAM	ab131607	Abcam	WB	1:500	Rabbit
NB100-56646	TTC11/FIS1	NB100-56646	Novus Biologicals	WB	1:500	Rabbit

Quantitative polymerase chain reaction (qPCR) analysis

Total RNA was extracted from frozen sections of the muscles using the RNeasy Plus Universal Mini Kit (QIAGEN, Hilden, Germany), and converted to complementary DNA with the help of reverse transcription random primers using the SuperScript IV VILO Master Mix (Thermo Fisher Scientific, Waltham, MA, USA), following the manufacturer’s instructions. Real-time PCR was performed using 10 ng of cDNA template for each gene analysis and quantified using an Applied Biosystems QuantStudio3 real-time PCR system (Thermo Fisher Scientific, Waltham, MA, USA). SYBR Green probes and primers were obtained from Takara Bio (Takara Bio, Otsu, Shiga, Japan) (Table 2). Lamin B receptor (*Lbr*) was used as an internal control, and gene expression levels were calculated using the 2−ΔΔCT relative quantitation method.

**Table 2:** Real-time reverse transcriptase-PCR primer sequences. PCR: polymerase chain reaction; Lbr: lamin B receptor.

Gene	Forward primer	Reverse primer
Myh1	5′-TGTGGACAAACTGCAATCAAAGG-3′	5′-CTGGATCTTGCGGAACTTGG-3′
Myh2	5′-TCAGGCTTCAAGATTTGGTGGAT-3′	5′-GCAGCTTGCGGAACTTGGA-3′
Myh4	5′-GCGACCTCAATGAAATGGAAATC-3′	5′-CTTTCAAGTCATCCTGGCCTCTG-3′
Hif1a	5′-TCTAGTGAACAGGATGGAATGGA-3′	5′-TCGTAACTGGTCAGCTGTGGTAA-3′
Epas1	5′-CGCCTCATGTCTCCATGTTCA-3′	5′-CCAGCTGGCGCTTTAGCTTC-3′
Ppargc1a	5′-CACCGTAAATCTGCGGGATG-3′	5′-TATCCATTCTCAAGAGCAGCGAA-3′
Atp2a1	5′-TCATTGCTCGGAACTATCTGGA-3′	5′-GCTGAAGACGCCTTGCCATTA-3′
Atp2a2	5′-GGTCAGTCTTAACGGCAGTGTG-3′	5′-CCCAAGCTCAGTCATGCAG-3′
Lbr	5′-GCTTCAACCACATCCTGCCTTA-3′	5′-TGGTGTTCATCACGGGCTTC-3′

Western blot analysis

Samples from cryosections of GH and MAS muscles were homogenized in the sample buffer solution with radioimmunoprecipitation assay buffer containing protease and phosphatase inhibitors and centrifuged at 15,000 rpm at 4 °C for 5 min. Then, 30 µg of proteins for each sample was loaded onto sodium dodecyl sulfate-polyacrylamide gel electrophoresis (SDS-PAGE) gels and blotted onto a polyvinylidene difluoride (PVDF) membrane. The blots were incubated with primary antibodies against ATP5A1, TTC11 (FIS1), MFN1, MFN2, PGC1α, OPA1, NADH:ubiquinone oxidoreductase complex assembly factor 1, mitochondrial transcription factor A, and glyceraldehyde 3-phosphate dehydrogenase (GAPDH) (Table 1). Horseradish peroxidase-conjugated secondary antibodies (Thermo Fisher Scientific, Waltham, MA, USA) were used for chemiluminescence detection. All bands were detected using Clarity Western ECL Substrate (Bio-Rad, Hercules, CA, USA) and visualized with the Image Lab 5.0 software (Bio-Rad, Hercules, CA, USA). All data were normalized to GAPDH and analyzed as relative band intensities using Image Lab 5.0 software.

Statistical analysis

Data are shown as mean ± standard deviation and analyzed with the Shapiro-Wilk normality test and a Welch’s t-test. Statistical significance was considered when the p-value was lower than 0.05. All statistical analyses were performed using IBM SPSS Statistics 22.0 (Chicago, IL, USA).

## Results

Chronic IH-induced changes in maternal blood oxygen saturation during pregnancy

Chronic IH-induced cyclical changes in maternal SpO_2_ levels responding to the IH cycle (3-min periods of 4-21% O_2_ in each chamber) are shown in Table 3. At baseline, IH mother rats showed stable SpO_2_ levels, similar to normoxic mother rats. All pups from both groups were born naturally and had increased body weight after birth and weaning. There was no statistical difference in the body weights of rats in the N/N and IH/N groups (the N/N group, 114.5 ± 5.9 g; the IH/N group, 106.4 ± 14.9 g) at day 35 after birth.

**Table 3:** Blood oxygen saturation (SpO2) of IH and normoxic pregnant rats at gestation day 20. IH: intermittent hypoxia.

	Baseline	IH cycle	
		Hypoxia	Reoxygenation
IH pregnant rats (n = 3)	96.3 ± 1.5 (%)	67.3 ± 6.6 (%)	97.4 ± 1.3 (%)
Normoxic pregnant rats (n = 3)	95.9 ± 0.6 (%)	N/A	N/A

Characteristics of GH and MAS muscles in offspring rats exposed to gestational IH

**Figure 1:**
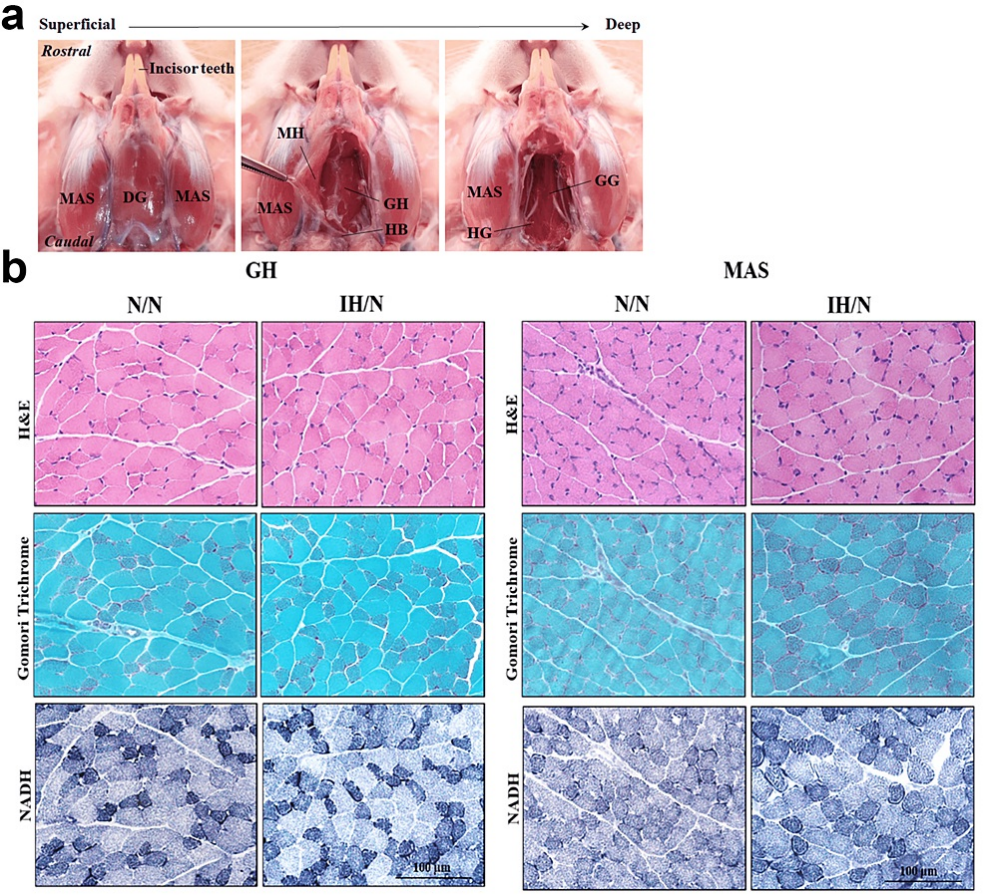
Anatomical location and histological images of the GH and MAS muscles of gestational IH offspring. (a) Ventral image of rat GH and MAS muscles. (b) The upper panel: hematoxylin & eosin stain; the middle panel: modified Gomori trichrome stain; the lower panel: NADH stain. Data represent male offsprings (n = 6) in each group. Scale bar: 100 μm. NADH: nicotinamide adenine dinucleotide reductase; MAS: masseter muscle; DG: digastric muscle; MH: mylohyoid muscle; GH: geniohyoid muscle; HB: hyoid bone; GG: genioglossus muscle; HG: hyoglossus muscle; IH: intermittent hypoxia; H&E: hematoxylin & eosin.

The muscle fiber histological images (Figure 1a, 1b) showed that both GH and MAS muscles were comparable between IH/N and N/N rats at the age of five weeks.

**Figure 2:**
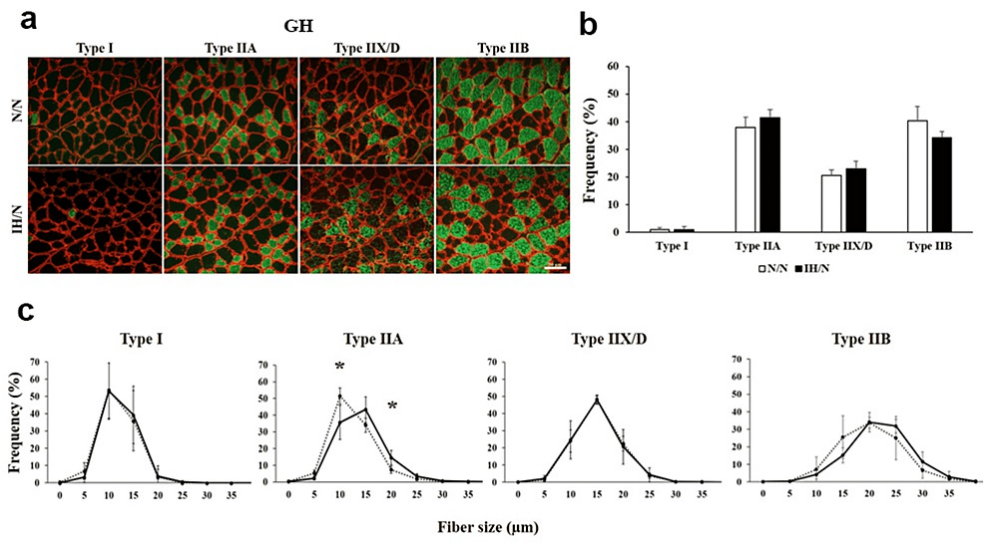
Distribution pattern of muscle fiber type in the GH muscle of gestational IH offspring rats. (a) Fiber type-specific immunohistochemical staining for type I, type IIA, type IIB, and type IID fibers with skeletal muscle membrane protein, laminin (red). Each panel shows a cross-sectional image of the GH muscle. Green areas indicate immuno-positive muscle fibers. (b) The graph indicates the percentage of muscle fiber type distribution in GH muscle from each group. (c) Histogram of the fiber size distribution of each muscle fiber type. Solid line and dotted line show N/N and IH/N groups, respectively. Data represent male offspring (n = 6) in each group. Scale bar: 50 μm. *p < 0.05 vs. the N/N group. GH: geniohyoid, IH: intermittent hypoxia.

**Figure 3:**
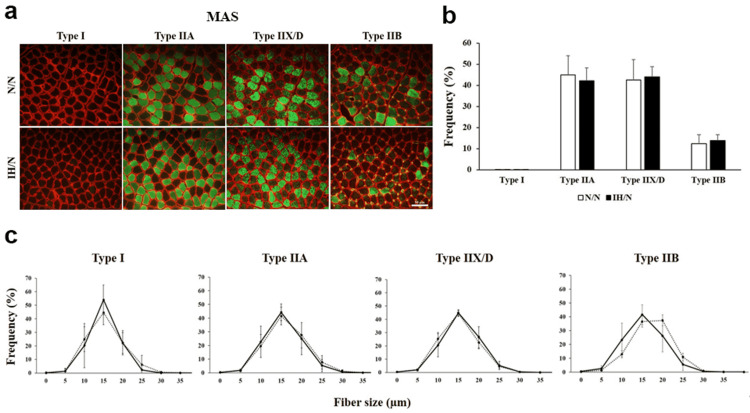
Distribution pattern of muscle fiber type in the MAS muscle of gestational IH offspring rats. (a) Fiber type-specific immunohistochemical staining for type I, type IIA, type IIB, and type IID fibers with skeletal muscle membrane protein, laminin (red). Each panel shows a cross-sectional image of the MAS muscle. Green areas indicate immuno-positive muscle fibers. (b) The graph indicates the percentage of muscle fiber type distribution in MAS muscle from each group. (c) Histogram of the fiber size distribution of each muscle fiber type. Solid line and dotted line show N/N and IH/N groups, respectively. Data represent male offspring (n = 6) in each group. Scale bar: 50 μm. MAS: masseter muscle, IH: intermittent hypoxia.

Skeletal muscle fiber type is characterized by slow fiber type to fast fiber type (type I→IIA→IIX/D→IIB). Among type II fibers, type IIA fibers have a higher oxidative capacity and fatigue more slowly than type IIX/D and IIB [16]. Immunohistochemically, both GH and MAS muscles consist predominantly of fast-type fibers and only a few slow-type fibers (Figure 2a, 3a).

The baseline fiber type proportion was heterogeneous between both muscles in N/N rats. There was no significant difference in the MAS muscle between the N/N and IH/N groups, while the GH muscle tended to decrease type IIB (fast-glycolytic) fibers in the IH/N group compared to the N/N group, but the difference was not statistically significant (Figure 2b, 3b). Moreover, a smaller diameter of type IIA fiber was observed in the GH muscle of the IH/N group compared to that of the N/N group, while the other fiber types were comparable between both groups (Figure 2c). In the MAS muscle, there were no significant differences in the fiber size of each fiber type (Figure 3c). Thus, histological data suggest that gestational IH decreases the size of type IIA fibers (oxidative fibers) in the GH muscle of the offspring, but the MAS muscle remains unaffected. 

Gestational IH-induced changes in genes related to fiber type characteristics in offspring rats

**Figure 4:**
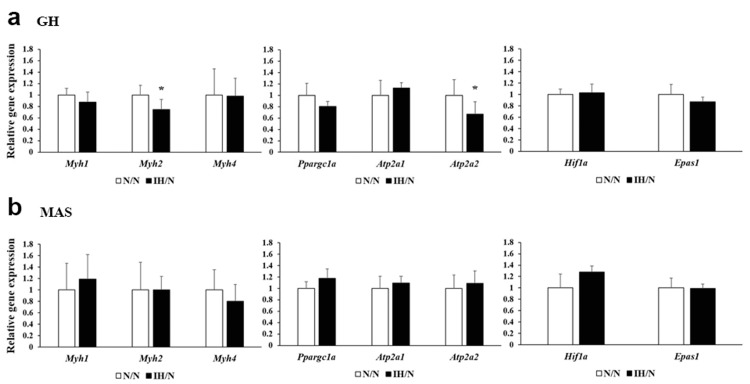
Quantitative polymerase chain reaction analysis in the GH and MAS muscles of offspring rats. Gene expression of muscle fiber type-related factors (*Myh1*, *Myh2*, *Myh7*, *Atp2a1*, and *Atp2a2*) and hypoxic-inducible factors (*Hif1a* and *Epas1*) in GH (a) and MAS (b) muscle from N/N and IH/N groups. The relative expression levels of each gene were normalized to the level of *Lbr *expression, and the relative expression levels were set to 1 for the N/N group. Data represent male offspring (n = 6) in each group. *p < 0.05 vs. the N/N group. GH: geniohyoid, IH: intermittent hypoxia, MAS: masseter muscle.

qPCR analysis confirmed that the expression of *Myh2* was significantly decreased only in the GH muscle of the IH/N group (Figure 4a).

In addition, Atp2a2 mRNA, encoding slow-type sarcoendoplasmic reticulum calcium ATPase (SERCA2), was significantly downregulated in the GH muscle of the IH/N group, in contrast to Atp2a1 mRNA (encoding first type-specific SERCA1). mRNA levels of both Atp2a1 and Atp2a2 were comparable in the MAS muscle between the IH/N and N/N groups. Hypoxia-inducible factors, HIF1 (encoded by Hif1a mRNA) and HIF2 (encoded by Epas1 mRNA), are often assessed in perinatal animal models of hypoxic neuronal injury and impaired responsiveness to hypoxia during postnatal life. In our experimental model, mRNA levels of Hif1a and Epas1 were comparable between the IH/N and N/N groups in the GH muscle (Hif1a, 1 ± 0.097 in the N/N group vs. 1.034 ± 0.151 in the IH/N group, p = 0.658; Epas1, 1 ± 0.179 in the N/N group vs. 0.871 ± 0.081 in the IH/N group, p = 0.140) (Figure 4a), and the MAS muscle also showed no significant differences in mRNA levels of the two hypoxic markers between the IH/N and N/N groups (Figure 4b). The GH qPCR data suggest that gestational IH affects mitochondrial biogenesis in the GH muscle.

Downregulation of mitochondrial biogenesis and fusion proteins in the GH muscle of gestational IH offspring

**Figure 5:**
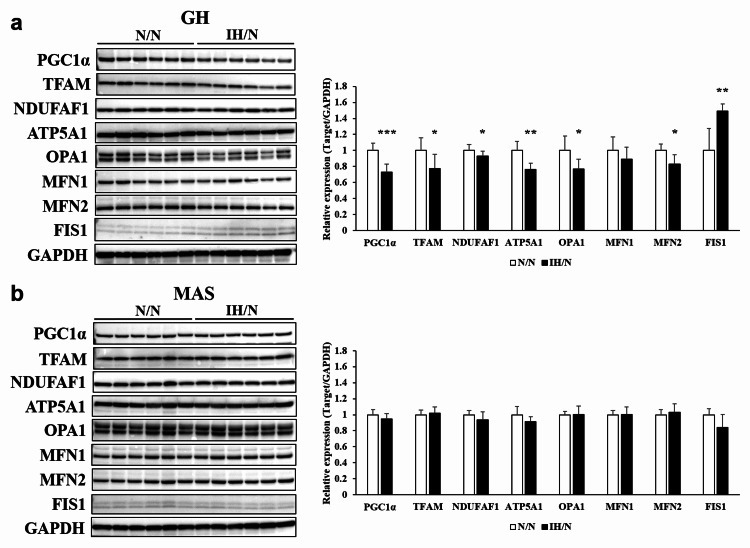
Protein levels of mitochondrial metabolic markers in the GH and MAS muscles of offspring rats. Western blot images for PPAR-gamma coactivator 1 (PGC1α), mitochondrial transcription factor A (TFAM), NADH:ubiquinone oxidoreductase complex assembly factor 1 (NDUFAF1), ATP5A1, optic atrophy 1 (OPA1), mitofusin (MFN)1, MFN2, mitochondrial fission 1 (FIS1), and glyceraldehyde 3-phosphate dehydrogenase (GAPDH) in GH (a) and MAS (b) muscles. The molecular weight markers were represented in the left line. The expression levels of the long and short isoforms of OPA1 were quantified together. The expression levels of the long and short isoforms of OPA1 were quantified together. The relative expression levels of each protein were normalized to the level of GAPDH expression, and the relative expression levels were set to 1 for the N/N group. Data represent male offspring (n = 6) in each group. *p < 0.05, **p < 0.01, and ***p < 0.001 vs. the N/N group. GH: geniohyoid; MAS: masseter muscle.

Western blot analysis revealed that gestational IH-induced significant downregulation of PGC1α protein in the GH muscle but not in the MAS muscle (Figure 5a, 5b).

The levels of mitochondrial fusion proteins, such as OPA1 and MFN2, were significantly decreased and the level of mitochondrial fission protein, FIS1, was significantly increased in the GH muscle of the IH/N group (Figure 5a). Data were supported by decreased levels of ATP synthase subunit alpha (ATP5A1) and mitochondrial transcriptional factor A (TFAM). In contrast, mitochondria-related protein levels were comparable in the MAS muscle between the N/N and IH/N rats (Figure 5b). These data suggest the possibility of impaired mitochondrial biogenesis and energy metabolism of the GH muscle in the offspring rat exposed to gestational IH, compared to the MAS muscle.

## Discussion

In utero exposure to environmental stress impairs the regulation of mitochondrial dynamics in the rat placenta and skeletal muscles of the offspring, with a reduction in transcriptional regulators (PGC1α and PGC1β) [10]. However, it is unknown about the effects of gestational IH on the postnatal development of the GH muscle, which has a key role in suckling, a motor behavior that then gives way to chewing in young offspring.

The GH muscle has only a few type I muscle fibers (only 1.0% in total muscle fibers from our analysis) and has a low aerobic capacity owing to a high percentage (98-100%) of type IIA, IIX/D, and IIB muscle fibers [17]. Our data were consistent with the findings of a previous study on the muscle fiber composition of rat GH muscle [17]. Slower muscle fibers have a higher concentration of mitochondria than fast fibers, and type IIA fibers have a higher concentration of mitochondria than fast fibers and type IIX/D and IIB fibers [18]. In our model, a decreased fiber diameter of type IIA fibers was observed in the GH muscle of the gestational IH group. Myh2 is a specific maker gene for type IIA fiber, and qPCR analysis suggests that gestational IH leads to small type IIA fiber via decreased Myh2 expression. Reduction of type I and/or type IIA fiber size correlates with decreased mitochondrial oxidative enzyme activity in muscle fibers [19]. Alteration in the diameter of type IIA fibers may reflect mitochondrial metabolism in the GH muscle of offspring of rats exposed to gestational IH.

PGC1 regulates energy metabolism through mitochondrial biogenesis. Western blot analysis showed that the protein level of PGC1α decreased in the GH muscle of preadolescent rats exposed to gestational IH. In our model, a subunit of the mitochondrial ATP synthase protein, ATP5A1, was also decreased in the GH muscle of the offspring of rats exposed to gestational IH. Silencing of the PGC1α gene induces mitochondrial fragmentation by decreasing the levels of OPA1, MFN1, and MFN2 and increasing the levels of mitochondrial fission proteins [9]. We observed decreased levels of OPA1 and increased levels of FIS1 in the GH muscle of the offspring with gestational IH. Mitochondrial fission separates the dysfunctional or damaged components from the healthy mitochondrial network [20]. However, excessive mitochondrial fission generates isolated mitochondria that are less efficient in ATP production and are dysfunctional because, under defects in mitochondrial fusion, they consume ATP to maintain their membrane potential [21]. Increased fission and/or decreased fusion leads to dysfunctional fragmented organelles, which results in decreased muscle fiber size and metabolic shifting [22]. Moreover, we observed the decreased protein levels of TFAM in the GH muscles of IH offspring, which reflects the decrease in mitochondrial biogenesis. Our findings suggest that gestational IH prompts muscular mitochondrial fission in the GH muscle, which leads to a decline in skeletal muscle mitochondrial function.

Postnatal IH causes mitochondrial fission by decreasing the expression of Mfn2 and increasing the expression of mitochondrial fission protein in cardiomyocytes, which leads to left ventricular hypertrophy and impaired contractile function in male rats [23]. Thompson et al. showed that gestational IH decreases cardiac contractile function in the heart of a male offspring due to the reduction of mitochondrial maximal respiration, respiratory reserve capacity, and complex IV activity rates in cardiomyocytes [24]. Gestational hypoxia during the advanced stages of fetal development decreases the mRNA levels of *Mfn2 *and* Pgc1α* and increases *Fis1 *and *Drp1* mRNA levels in the heart of the rat offspring, which show mitochondrial structural abnormalities, dysfunction, decreased biogenesis, and mitochondrial fission/fusion imbalance [25]. Our findings suggest that gestational IH induces mitochondrial fission/fusion imbalance and impairment in the GH muscle of offspring rats.

The MAS muscle was insusceptible to gestational IH, unlike the GH muscle. We observed specific type distribution, fiber size, and frequency of type I, IIA, IIX/D, and IIB in rat GH and MAS muscles at baseline. Gestational IH offspring showed a change in fiber size of type IIA fibers of the GH muscles, in contrast to the MAS muscle. Fogarty and Sieck showed different force and fatigue properties between intrinsic (superior and inferior longitudinal and transversalis) and extrinsic (genioglossus) muscles in the rat tongue in relation to muscular fiber type percentage, and they indicated that the rat genioglossus muscle is more fatigue resistant due to a higher proportion of type I and IIA fibers than the intrinsic tongue muscles [26]. Their demonstration based on muscular fiber type percentage may provide one possible explanation for the different metabolic responses of the GH and MAS muscles to gestational IH. Moreover, functional development of the MAS and GH muscles occurs after birth, which may elicit different responses of each muscle in the offspring. The development of rodent MAS muscles is closely associated with facial development and feeding after birth [27].

The MAS and GH muscles have different embryonic origins that arise at spatially distinct locations during the early embryonic period. A previous study on orofacial muscle formation in mouse embryos has shown the initiation of trigeminal innervation to the MAS muscle at embryonic day (ED) 10.5, in the first pharyngeal arch and a significant change in the MHC composition of the embryonic MAS muscle from EDs 14 to 18 [27]. On the other hand, the myogenic cells of the murine tongue start to migrate a long distance from the occipital somites toward the pharyngeal arches around ED 10.5 and form tongue muscles [28]. Moreover, hypoxia and increased mitochondrial respiration, with the concomitant production of reactive oxygen species, are known to affect morphogenic processes and cell function during embryonic development [29]. Early fetal hypoxia causes tissue-and-cell type-specific growth restriction and cell proliferation in murine embryonic cells of the myocardium but not in the cells of the spinal cord and brain [30]. In this study, the IH period from EDs 7 to 20 covered the period of early embryonic development for the MAS and GH muscles in rodents. The susceptibility of MAS and GH muscles to gestational IH may depend on tissue-specific effects of hypoxia on myogenic cells, which have different origins and formation patterns during early embryo development.

## Conclusions

Gestational IH induces mitochondrial impairment in the GH muscle of the male offspring but not in the MAS muscle. These results suggest the different susceptibility of prenatal IH to mitochondrial metabolism of the GH muscle in male offspring rats compared to the MAS muscle.
